# Increased abundance of *Limosilactobacillus reuteri* in the gut of selectively bred high-tameness mice and its association with behavioural changes

**DOI:** 10.1093/dnares/dsag006

**Published:** 2026-06-25

**Authors:** Bhim B Biswa, Hiroshi Mori, Atsushi Toyoda, Kazumichi Fujiwara, Ken Kurokawa, Tsuyoshi Koide

**Affiliations:** Mouse Genomics Resource Laboratory, National Institute of Genetics, Mishima, Shizuoka 411-8540, Japan; Graduate Institute for Advanced Studies, SOKENDAI, Mishima, Shizuoka 411-8540, Japan; Graduate Institute for Advanced Studies, SOKENDAI, Mishima, Shizuoka 411-8540, Japan; Genome Diversity Laboratory, National Institute of Genetics, Mishima, Shizuoka 411-8540, Japan; Comparative Genomics Laboratory, National Institute of Genetics, Mishima, Shizuoka 411-8540, Japan; Mouse Genomics Resource Laboratory, National Institute of Genetics, Mishima, Shizuoka 411-8540, Japan; Graduate Institute for Advanced Studies, SOKENDAI, Mishima, Shizuoka 411-8540, Japan; Genome Evolution Laboratory, National Institute of Genetics, Mishima, Shizuoka 411-8540, Japan; Mouse Genomics Resource Laboratory, National Institute of Genetics, Mishima, Shizuoka 411-8540, Japan; Graduate Institute for Advanced Studies, SOKENDAI, Mishima, Shizuoka 411-8540, Japan

**Keywords:** gut microbiota, domestication, wild heterogeneous stock, lactic acid bacterium, oxytocin

## Abstract

Domestication alters animal behaviour, particularly tameness. We previously established 2 tamed mouse groups by selective breeding for active tameness–defined as the motivation to approach a human hand–from genetically heterogeneous wild-derived mouse stock, together with 2 nonselected control groups. Genetic analyses identified loci associated with active tameness, but their low heritability suggested contributions from nongenetic factors. We therefore hypothesized that the gut microbiota, which has been shown to influence brain function, contributes to behavioural changes associated with active tameness. To test this hypothesis, we conducted shotgun metagenomic analyses of faecal samples from 10 males and 10 females (80 individuals total) from the 2 tamed and 2 nonselected groups. Tamed mice exhibited markedly higher levels of active tameness, accompanied by elevated blood concentrations of oxytocin and pyruvate. While overall taxonomic and functional diversity of the gut microbiota was largely unchanged, the abundance of *Limosilactobacillus reuteri* was significantly increased in the tamed mice. Administration of a pyruvate-secreting *L. reuteri* strain to nonselected mice elevated blood oxytocin levels and enhanced active tameness, although plasma pyruvate levels were not increased. These findings suggest that *L. reuteri* is associated with behavioural modulation, potentially via oxytocin-related pathways, and provide mechanistic insight into microbial contributions to animal domestication.

## 1. Introduction

Domestication is a sustained multigenerational process by which captive animals adapt to humans and their environment. Several factors contribute to animal adaptation in captivity. While most adaptation is due to genetic changes, other factors like experiences, human interaction^1–3^, temperature^4–6^, and food^7^ also play a role. Domestication involves both genetic changes across generations and environmentally induced events^8^. Studies have shown that gut microbiota can influence host behaviour, brain development, and cognition by altering metabolic pathways or affecting the immune system^9,10^. Research has explored the link between gut microbiota and domestication in various animals, identifying bacteria that may influence domestication^11–17^. However, these studies face limitations such as contamination risks, lack of common ancestry, differences in diet, crossover contamination, and antibiotic use.

These challenges can be mitigated by using animals bred in a controlled setting with a singular focus on phenotypic selection and well-documented lineages. The wild-derived heterogeneous stock (WHS) mice, which have been selectively bred for tameness, are a good example of this approach. Tameness is a behavioural phenotype necessary for animals to adapt to humans and presents in 2 types: the motivation to approach humans (active tameness) and a reluctance to avoid humans (passive tameness). To increase active tameness, mice that exhibited a relatively high motivation to approach humans in the active tameness test, which evaluates levels of active tameness in mice^18^, were bred selectively using WHS mice. A founder group of WHS mice was developed by crossing 8 different wild inbred strains originating from different countries and representing 3 subspecies groups of *Mus musculus domesticus, castaneus, and musculus*^19,20^. A group of WHS was bred with 16 pairs of mice in each generation to maintain high genetic diversity. Four groups of WHS were generated from the common founder WHS group by splitting them into 2 selected (S1 and S2) and 2 nonselected WHS groups (C1 and C2). Genetic components of active tameness have been reported in WHS mice, indicating that genetics significantly influence tameness^20^. Previous genetic analyses identified 2 adjacent but distinct loci associated with active tameness on chromosome 11. However, the relatively low estimated realized heritability suggests that nongenetic factors may also contribute to differences in active tameness between selected and nonselected groups^20^. We therefore hypothesized that the gut microbiota, which has not yet been explored in this system, may contribute to behavioural variation associated with active tameness. All WHS mice were housed in the same animal room, but in separate breeding racks. Both selected and nonselected groups were kept under specific pathogen-free conditions, with radiation-sterilized food and ultrafiltration-sterilized water. Thus, any gut microbiota differences between groups can be directly attributed to host selection pressure. In our study, to characterize the gut microbiota in WHS mice, we conducted metagenomic analysis using genomic DNAs obtained from the faeces of animals from the selected and nonselected groups. An intervention strategy was then used to introduce specific bacteria into the drinking water of the mice to examine changes in their tameness behaviour.

## 2. Materials and methods

### 2.1. Experimental conditions and mice

All experiments in this study were conducted in strict accordance with the guidelines and protocols sanctioned by the ‘Committee for Animal Care and Use’ at the National Institute of Genetics (permit numbers: R4-25 and R5-7). The mice were provided with a standard chow diet, CE-2 (CLEA Japan, Inc., Tokyo, Japan), which was radiation-sterilized at 15 KGy. Both food and ultrafiltration sterilized water (supplemented with 3 ppm chlorine) were availed to the mice ad libitum. The mice were housed in a temperature-controlled room at 23 ± 2 °C with a humidity range of 50 ± 10% and a 12-h light-dark cycle with lights switched on at 6:00 AM. The first set of experiments was carried out using mice from the 27th generation of the WHS. The establishment of WHS mice and selective breeding has been described in previous papers^20,21^ and in the Supplementary Methods. The mouse stocks used in this study, S1, S2, C1, and C2, are available from the following website https://grc.nig.ac.jp/mgrlab/index.xhtml.

### 2.2. Tameness test

The tameness test was performed at 6 wk of age, as described in a previous study^18^ during the light period between 14:00 and 17:00. The test was conducted in an open-field arena (40 × 40 × 40 cm) made of grey polyvinyl chloride (O’Hara & Co. Ltd, Tokyo, Japan) with the floor illuminated with approximately 100 lux at the centre^22^. The tameness test was a 3-min protocol divided into 1 min each of the active and passive tameness tests and 3 trials of the stay-on-hand test. In the active tameness test, the experimenter's hand was kept 10 cm away from the test mouse for 1 min with the fingers moving slightly and rhythmically to observe the animal's motivation to approach and contact the human hand. In the passive tameness test, the experimenter attempted to touch the mouse with their hand, and mouse behaviour was recorded for 1 min. This test examined how long the mice could tolerate being touched by a human hand. In the stay-on-hand test, each mouse was picked from its tail using silicon-coated tweezers, placed on the experimenter's hand, and stroked gently until it attempted to get off the hand.

### 2.3. Tameness test parameter clustering

The analysis was performed in R Ver.4.3.2. The tameness test results for all 80 samples were normalized using ‘min-max’ normalization. Clustering was executed based on the correlation distance, employing the Ward method via the ‘hclust’ function. The statistical significance of the identified clusters was assessed using pvclust Ver.2.2.0^23^, setting the significance level at 5% for each clustering analysis.

### 2.4. Metagenomic DNA isolation from mice faeces

In our modified approach to metagenomic DNA isolation, we utilized protocol #6 from Costea *et al*.^24^, incorporating the QIAamp Fast DNA Stool Mini Kit (QIAGEN GmbH, Hilden, Germany) for DNA extraction. The detailed protocol is provided in the Supplementary Methods.

### 2.5. Plasma metabolites analysis

For metabolomic analysis, samples were analysed by Human Metabolome Technologies, Inc. (https://humanmetabolome.com/jpn/). A basic scan was performed to detect more than 1000 water-soluble metabolites. The protocol for this analysis has been described previously^25–28^. Briefly, metabolome analysis (CE-TOFMS) was performed on 12 randomly selected mouse plasma samples in 2 modes for cationic and anionic metabolites.

### 2.6. *Limosilactobacillus reuteri* isolation

Caecum and faecal samples were collected from mice of selected group (S1), mixed, and then serially diluted. This diluted mixture was cultured on *Limosilactobacillus reuteri* Isolation Media (LRIM)^29^ agar plates, with raffinose (Tokyo Chemical Industry Co. Ltd., Tokyo, Japan) as the sole carbon source. The plates were incubated for 48 h at 45 °C under anaerobic conditions (5% CO_2_). To maintain the anaerobic condition, plates were stored in a sealed box containing 1 AnaeroPack sachet (Mitsubishi gas chemical company, Inc., Tokyo, Japan). For the isolation of pure cultures, selected colonies were first streak plated twice on LRIM plates. This was followed by third streak plating on De Man–Rogosa–Sharpe (MRS) (Merck-Millipore, MA, United States) agar plate.

### 2.7. *L. reuteri* phylogenetic analysis

All isolated colonies were cultured in MRS media, after which DNA was extracted using the NucleoSpin Microbial DNA kit (Takara Bio Inc., Shiga, Japan), according to the manufacturer's protocol. Primer pairs from previous research were used^30,31^ to amplify 16s rDNA followed by sanger sequencing (Supplementary Methods). Taxonomic identification of obtained sequences was done using NCBI BLASTn^32^. To infer phylogeny, along with our sequences, we first obtained 16S rRNA gene sequence of multiple *Limosilactobacillus* species from NCBI along *Lactobacillus helveticus* as outgroup. All sequences were aligned using the MAFFT online server with ‘FFT-NS-I’ command^33,34^. Using Model finder^35^, we found ‘TPM3u + F + I’ as the best-fit model for our data. We used IQ-TREE 2^36^ with 1000 bootstrap replicates to construct a maximum likelihood tree. This tree was edited using iTOL online server^37^.

### 2.8. *L. reuteri* administration through drinking water

PBS (vehicle) or bacteria (∼1 × 10^8^ CFU/Cage/d) was added to the drinking water of the mice each day between 17:00 and 18:00 (Supplementary Methods). *L. helveticus* (JCM 1120) was provided by the RIKEN BRC through the National BioResource Project of the MEXT, Japan and used as a control species. Mice from the 37th generation of C1 group were used. The administration of *L. reuteri* began immediately postweaning, at approximately 3 wk of age, and was continued for 21 d. To minimize isolation stress, mice were housed in same-sex pairs. Faecal samples were collected from each individual both before the start and at the end of the experiment. In addition, we monitored and recorded the daily water consumption of each cage and measured the body weight of the mice at the end of the experiment. After the 21-d bacterial administration period, a tameness test was conducted. This was followed by the collection of faecal and plasma samples for further analysis.

### 2.9. Quantitative PCR quantification of *L. reuteri* and *L. helveticus*

For quantification of bacteria from mouse faeces, quantitative PCR (qPCR) was conducted using published primers^38–41^. Details can be checked in Supplementary Methods. The qPCR analysis was performed in triplicates (technical replication).

### 2.10. Mice serum pyruvate and oxytocin concentrations

Blood serum concentrations of pyruvate and oxytocin was measured using biochemical assay and ELISA kit respectively. Details can be checked in Supplementary Methods. Both assays were performed in duplicates (technical replication). Because *L. helveticus* JCM1120 was included only as a pyruvate-secreting control strain, oxytocin was not assayed in this group.

### 2.11. Shotgun metagenomic sequencing

Library preparation and sequencing were performed at the Advanced Genomics Center of the National Institute of Genetics. Briefly, genomic DNA was fragmented to an average size of 500 bp using a DNA shearing system (M220 focused ultrasonicator; Covaris Inc., MA. United States). A paired-end library was constructed using a TruSeq DNA PCR-Free Library Prep kit (Illumina, CA, United States) and size-selected on an agarose gel using a Zymoclean Large Fragment DNA Recovery Kit (Zymo Research, CA, United States). The final library was subjected to paired-end sequencing using an Illumina NovaSeq 6000 sequencer with a read length of 150 bp.

### 2.12. Bioinformatics analysis of metagenomic sequence data

#### 2.12.1. Quality control of metagenomic sequences

All sequencing reads obtained were quality-filtered using KneadData (Ver.0.10.0) (https://github.com/biobakery/kneaddata), where low-quality sequences and any contamination from PhiX, host (mouse_C57BL_6NJ.1), human genome (hg37dec_v0.1.1), or transcriptome (human_hg38_refMrna.1) were discarded. KneadData uses Trimmomatic (Ver.0.33)^42^ to remove adapters, TRF^43^ to remove repetitive sequences, and Bowtie2 (Ver.2.4.5)^44^ to remove contaminants.

#### 2.12.2. Taxonomic analysis

Taxonomic analysis of the gut microbiome was conducted using Kraken 2 (Ver.2.1.2)^45^. We employed the full NCBI/RefSeq database made by Wright et al. (NCBI RefSeq Complete V205 (1189 GB))^46^ to ensure comprehensive species identification within the gut samples. Default parameters were used for Kraken 2, with a confidence score threshold set at 0.15. This threshold was chosen to strike a balance between the robustness of species identification and maximizing the percentage of sequences utilized in the analysis. Further refinement of the Kraken 2 output was achieved through Bayesian re-estimation using Braken2 (Ver.2.6.2), also utilizing its default settings^47^.

#### 2.12.3. Functional analysis

For functional analysis in our study, we utilized HUMAnN3 (Ver.3.0.1)^48^ employing the ‘–bypass-prescreen’ option. We concatenated the filtered forward and reverse sequences from each sample and mapped them to the comprehensive ChocoPhlAn database (Ver.201901b). Sequences that remained unassigned after this step were further analysed against the UniRef90 database. To identify specific metabolic pathways, we employed the MetaCyc database^49^. We obtained Copies per Million data for each gene family and pathway using the ‘humann_renorm_table’ command.

#### 2.12.4. Abundance and diversity analysis

Diversity analysis and graph generation in our study were carried out using the MicroEco package^50^ We normalized the abundance data obtained from Braken2 by rarefying it to the size of the smallest sample^51^. To assess the statistical significance of alpha diversity, we employed the Kruskal–Wallis Rank Sum test. Beta diversity was analysed based on Bray-Curtis dissimilarity; analysis was complemented by principal coordinate analysis, which was applied to both taxonomic and functional data. In our correlation analysis, we first conducted a random forest classification using ‘randomForest’ package^52^, utilizing mouse group information (C1, C2, S1, and S2) on the normalized Bracken2 bacterial abundance data, adjusting the p-value using the Benjamini-Hochberg False Discovery Rate (FDR) method. Afterward, we normalized the scores of tameness parameters through min-max normalization. These normalized tameness scores were then subjected to Pearson correlation with the top 40 significant taxa identified in the random forest analysis using ‘pheatmap’ package^53^.

#### 2.12.5. MaAsLin2 association analysis

For association between taxonomic abundance with each mice group, we use Bracken2 abundance data. Initially it was normalized using ‘Total sum scaling’, then transformed into a log scale, after which association analysis was performed. In association analysis between functional pathways, we did not perform additional normalization or transformation as HUMAnN3's output was already normalized. In both analyses, we used a linear model and compared S1 and S2 groups to their respective controls.

#### 2.12.6. Metagenome-assembled genome (MAG) generation

Default settings were used in different software, unless otherwise specified. Filtered sequences were assembled using metaSPAdes (*k* = 21, 33, 55) (Ver.3.15.4)^54^ for a single assembly or MEGAHIT (–*k*-min 21 –*k*-max 77) (Ver.1.2.9)^55^ for co-assembly. Multiple binners with different binning strategies were used. The MEGAHIT assembly was binned using MetaCoAG (Ver.1.0)^56^, MaxBin2 (Ver.2.2.7)^57^, and MetaBAT2 (Ver.2.15)^58^. This was followed by the generation of a nonredundant set of bins using DAStool(search_engine diamond) (Ver.1.1.4)^59^. For the metaSPAdes-generated assembly, we utilized SemiBin(multi_easy_bin) (Ver.0.7.0)^60^ and VAMB(multisplit) (Ver.3.0.8)^61^. All bins produced were subjected to contamination filtering using MDMcleaner (Ver.0.8.3)^62^. Bins devoid of chimeric sequences, as confirmed by GUNC(Ver.1.0.5)^63^, and those with MAG quality scores above 0.5 were retained for further analysis. These filtered bins were then evaluated for contamination and completeness using CheckM (lineage_wf) (Ver.1.2.0)^64^. To create a nonredundant bin set, we employed the ‘dereplicate’ command of dRep (Ver.3.0.0) (-comp 50, -con 10, -pa 0.90, -sa 0.95, -nc 0.30, and -cm larger)^65^. Finally, the taxonomic classification of all bacteria and the generation of the species tree were performed using GTDB-Tk (classify_wf) (Ver.2.1.1) with the ‘r207_v2’ database^66^. MAGs phylogenetic tree was generated using FastTree (Ver.2.1.11)^67^ within GTDB-Tk using the ‘infer’ command. The generated tree was then refined and edited using the Interactive Tree of Life (iTOL) tool^37^.

#### 2.12.7. MAG analysis

To identify novel MAGs, we utilized the ‘compare’ command of dRep (Ver.3.0.0) (-pa 0.90 -sa 0.95 -nc 0.30 -cm larger)^65^. This analysis was conducted on a combined database that included the 1573 mouse gut MAGs previously compiled by Kieser *et al*.^68^ along with the 374 MAGs generated in our study. We classified our MAGs as novel if they did not cluster with any of the existing MAGs in this integrated database at 95% average nucleotide identity, thereby distinguishing them as unique at species level. To quantify the relative abundance of each MAG, we employed CoverM (Ver.0.6.1) (https://github.com/wwood/CoverM) and used minimap^69^ to map reads to our MAG database. All 27 novel MAGs were annotated using DFAST^70^.

#### 2.12.8. Sequencing and library preparation of *L. reuteri* strains

Genomic DNA of strains NIG-23 and NIG-A41 was extracted using Bactozol and DNAzol (Molecular Research Center, Inc. OH, United States) according to the manufacturer's instruction and subjected to RNase treatment followed by AMPure beads (Beckman Coulter, Inc. CA, United States) purification to remove residual RNA prior to library preparation. For long-read sequencing, libraries were prepared using the Native Barcoding Kit 96 V14 (SQK-NBD114.96; Oxford Nanopore Technologies, Oxford Science Park, UK). High-molecular-weight genomic DNA was first fragmented using a g-TUBE (Covaris, MA, United States), and fragments shorter than 25 kb were removed by size selection with BluePippin (Sage Science, MA, United States). The size-selected DNA was then used for library construction according to the manufacturer's protocol and sequenced on the PromethION platform (Oxford Nanopore Technologies). For short-read sequencing, libraries were prepared using the TruSeq DNA PCR-Free Library Prep Kit (Illumina). Genomic DNA was fragmented using a Focused-ultrasonicator M220 (Covaris), and libraries were constructed following the manufacturer's instructions. Size selection was performed by agarose gel extraction to obtain an insert size of approximately 500 bp. The libraries were sequenced on the NovaSeq 6000 platform (Illumina).

#### 2.12.9. Genome assembly and annotation

Nanopore long reads were assembled de novo using Flye v2.9.5^71^ with the parameter ‘–nano-hq’, specifying FASTQ-format high-quality Nanopore reads. The resulting assemblies were subsequently polished with Illumina short reads using Pilon v1.23^72^ with default parameters to correct base-level errors. Genome annotation was performed using Bakta v1.12^73^. Prior to annotation, each assembled genome sequence was rotated such that the dnaA gene was positioned at the beginning of the sequence, ensuring a standardized genomic coordinate system for downstream analyses. The predicted open reading frames annotated by Bakta were further functionally annotated using eggNOG-mapper v2.1.13^74,75^ to assign COGs^76,77^. During this step, the options ‘–itype proteins’ and‘–tax_scope bacteria’ were applied.

#### 2.12.10. Statistical analysis

Statistical analyses were conducted using GraphPad Prism Ver.10.1.2 and R Ver.4.3.2. Details of statistical tests are provided in each figure legend. A 2-way ANOVA with Tukey's test was used to assess the effect of sex on tameness behaviour. For statistical association analysis of plasma metabolites, taxonomic abundance, and functional abundance, MaAsLin2(Ver.1.12.0)^78^ was used with a Benjamini-Hochberg q-value of 0.25 following previous microbiome studies^79,80^.

## 3. Results

### 3.1. Selective breeding leads to increase in active tameness behaviour and blood oxytocin levels

**Fig. 1.**
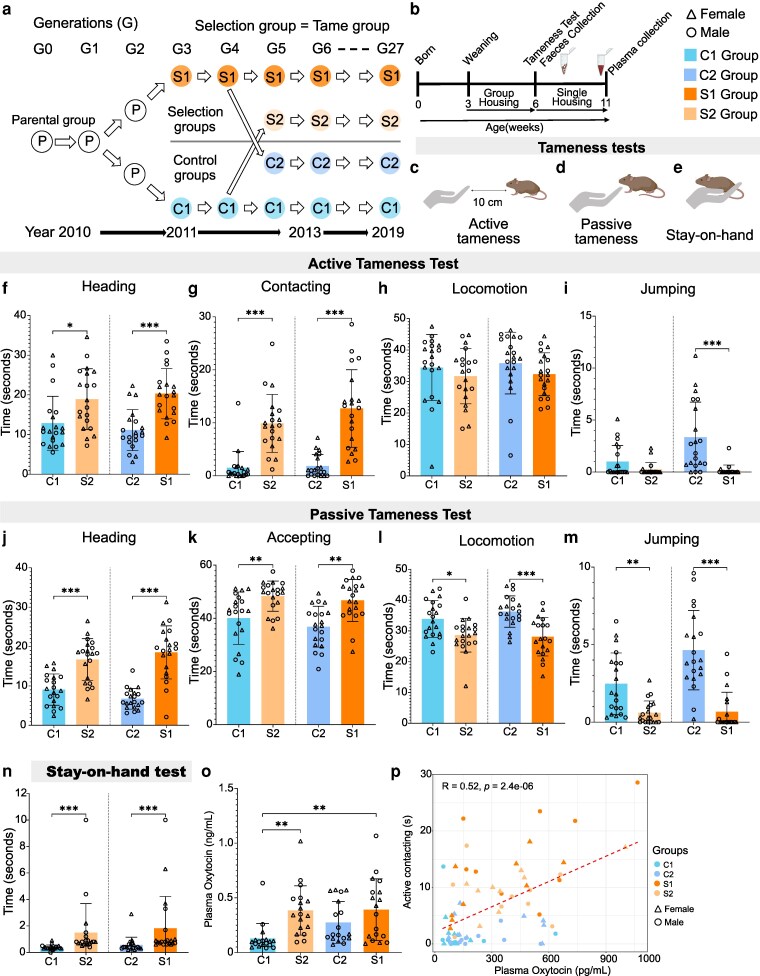
Tameness and plasma oxytocin concentration is higher in selected mice compared with control. a) Scheme of wild heterogeneous stock generation; b) Experimental timeline, c–e) tameness test illustrative depiction, c) active tameness test, d) Passive tameness test, e) stay-on-hand test, f–i) active tameness test results, f) heading, g) contacting, h) locomotion, i) jumping, j–m) passive tameness test results, j) heading, k) accepting, l) locomotion, m) jumping, n) stay on hand test, o) Plasma oxytocin concentration p) Pearson correlation between oxytocin concentration and active contacting time. N for tameness test = 80 (20 in each group with 10 male and 10 female); N for oxytocin ELISA = 72 (18 in each group with 9 male and 9 female). (*P < 0.05; **P < 0.01; ***P < 0.001). Bar graphs display means ± SD with individual data points. Tameness test data were assessed for normality using the Shapiro–Wilk test. For normally distributed data, 1-way ANOVA and Tukey's test for multiple comparisons were used. For non-normally distributed data, the Kruskal–Wallis test and Dunn's test were applied. Oxytocin concentration was analysed with 2-way ANOVA followed by Tukey's test.

At the 27th generation of breeding, we examined tameness-related behaviours in the selected and nonselected groups of mice (Fig. 1a). We conducted tests of active tameness, passive tameness, and stay-on-hand tests on 80 mice: 10 male and 10 female mice for each group (Fig. 1b–e). As S1 and C2, and S2 and C1 are genetically closely related (Fig. 1a), we compared the data between the 2 pairs to determine the effect of selective breeding on active tameness compared with the nonselected control, hereafter. Data on 9 behavioural parameters (Table S1)—heading, contacting, locomotion, and jumping in the active tameness test (Fig. 1f–i); heading, accepting, locomotion, and jumping in the passive tameness test (Fig. 1j–m); and median staying time in the stay-on-hand test (Fig. 1n)—were obtained. Given that there was no effect of sex in the 2-way analysis of variance (2-way ANOVA; Table S2), data from both sexes were combined in all tameness analyses. From the clustering analysis of all 9 parameters, we identified 2 significant clusters that categorize the parameters as either positively or negatively associated with tameness (Fig. S1). Regarding active contacting, a parameter of selective breeding, both S1 (*P* < 0.001) and S2 (*P* < 0.001) exhibited significantly longer contact durations compared with their respective controls (Fig. 1g). Other tameness-related parameters, such as heading in the active tameness test (Fig. 1f) (S1, *P* < 0.001; S2, *P* < 0.05), heading (Fig. 1j) (S1, *P* < 0.001; S2, *P* < 0.001) and accepting (Fig. 1k) (S1, *P* < 0.001; S2, *P* < 0.01) in the passive tameness test were higher in the selected groups than in the nonselected groups. In the stay-on-hand test, mice of the selected groups stayed on the hand longer than controls (Fig. 1n) (S1, *P* < 0.001; S2, *P* < 0.001). In contrast, parameters that are characteristic of wild mice, such as active jumping (Fig. 1i) (C2, *P* < 0.001), passive locomotion (Fig. 1l) (C2, *P* < 0.001; C1, *P* < 0.05), and passive jumping (Fig. 1m) (C2, *P* < 0.001; C1, *P* < 0.01), were higher in the nonselected groups than in the selected groups. Collectively, S1 and S2 showed higher active and passive tameness than the nonselected group.

Previous studies on WHS mice showed that selected groups exhibit higher level of social behaviours as well as higher expression of hippocampal *Oxtr*, a receptor gene for oxytocin which play crucial role in social bonding^81^ in these mice^20,82^. To see how selective breeding on tameness affect oxytocin pathway, we measured oxytocin levels in plasma collected from these mice. Results showed significantly higher oxytocin levels in both S2 and S1 compared with C1 (S1, *P* < 0.01; S2, *P* < 0.01). Although difference was not significant, S1 also showed higher oxytocin levels compared with the control C2 (Fig. 1o). Furthermore, a significant mild Pearson correlation (R = 0.52, *P* = 2.4 × 10^−6^) was observed between oxytocin levels and active contracting time (Fig. 1p), highlighting a possible link between oxytocin and tameness behaviour.

### 3.2. Gut microbiome repertoire in mice of selected and nonselected groups

**Fig. 2.**
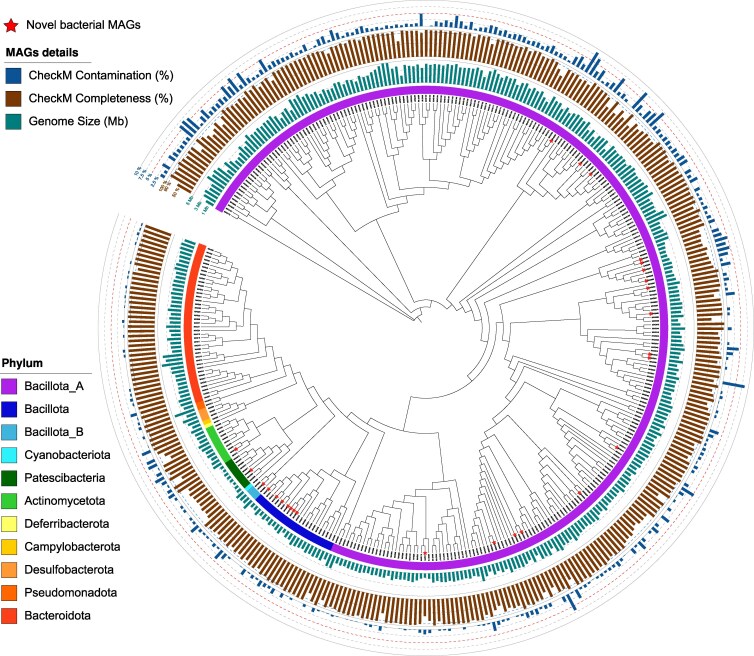
Cladogram of 374 MAGs inferred using GTDB output. The innermost circle shows the phylum assignment of each MAG. The second, third, and fourth circles indicate genome size (Mb), CheckM completeness (%), and CheckM contamination (%), respectively. Novel MAGs are indicated by star symbols.

To examine the gut microbiota of the selected and nonselected groups, shotgun metagenomic sequencing analysis was performed on 80 faecal samples collected from each mouse after the tameness test. On average, we obtained 31 million paired reads per sample. Metagenome-assembled genomes (MAGs) were used to analyse the gut microbiome composition of WHS mice. Because the gut microbiota is a complex flora, we employed 2 assemblers and multiple binning approaches to obtain as many good-quality MAGs as possible (Fig. S2A). Using these approaches, we generated 14 816 MAGs (details of MAGs obtained from each binner are shown Fig. S2B and C). After removing the redundant MAGs, we obtained 374 bacterial MAGs that were more than 50% completeness and had less than 10% contamination (Fig. 2, Fig. S2E and Table S3). Of these MAGs, 226 were high-quality as they were more than 90% completeness and less than 5% contamination (Fig. S2D). These 374 MAGs spanned 11 phyla, with 281 MAGs belonging to the *Bacillota A* phylum (Fig. 2 and Table S3). There are multiple mouse metagenome catalogues available^83,84^, with the most recent being the Mouse Gastrointestinal Bacteria Catalogue^85^ and the Comprehensive Mouse Microbiota Genome Catalogue (CMMGC)^68^. For our analysis, we used the CMMGC to compare our MAG data because it covers a higher number of species. Upon comparison, we identified 27 species level novel MAGs that have not been previously reported. This is further confirmed by ‘taxonomic check’ in DFAST^70^. Of these 27 MAGs, 1 each belonged to *Actinomycetota*, *Bacillota B*, and *Patescibacteria*, 8 to *Bacillota*, and 16 to *Bacillota A* (Fig. 2). Additionally, despite the efficacy of machine learning binners in deriving higher quantity and quality MAGs, their limitations in binning all bacteria types were identified, suggesting that the application of combined methodology that integrates both single assembly and co-assembly, as well as the application of multiple binning tools is advantageous (Fig. S2E and F).

**Fig. 3.**
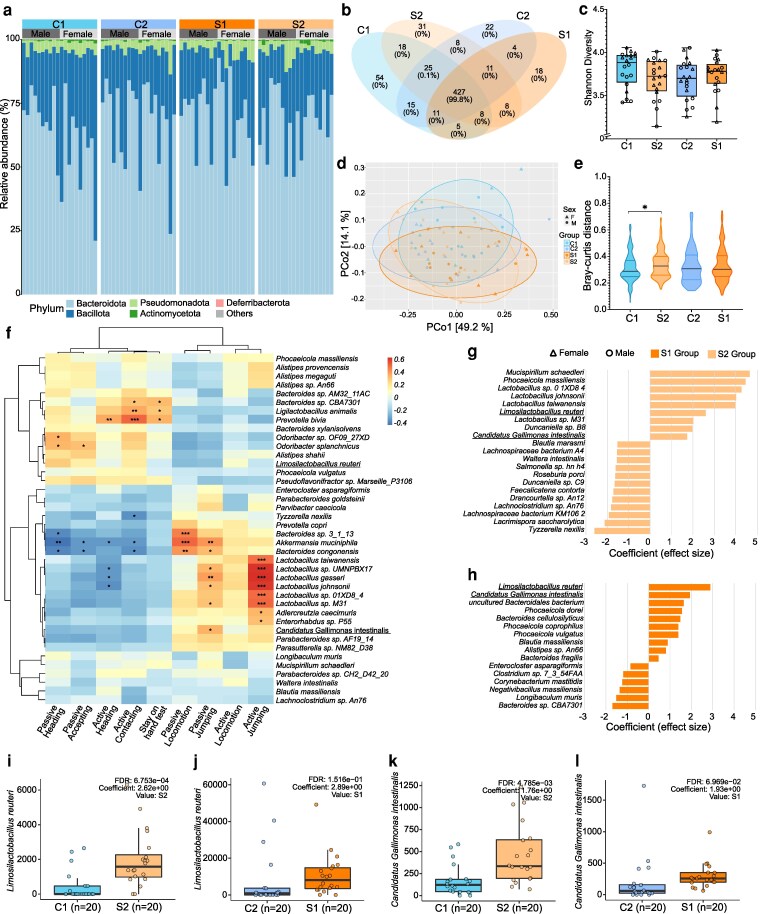
Gut microbiome diversity is similar in all groups. a) Phylum level relative abundance in all groups, 5 most abundant phylum is shown; full dataset includes 11 phyla, 21 classes, 45 orders, 103 families, and 339 genera, b) Ven diagram of 665 bacterial species present in WHS mice gut, c) Shannon diversity, d) Beta diversity based on Bray–Curtis dissimilarity, e) Bray curtis distance between each groups, f) Hierarchical clustering using Pearson correlation distance between top 40 significantly different taxa (result of random forest analysis) with score of parameters of tameness test. Significant correlation is marked with ‘*’ g) Association analysis in MaAsLin2 between S2 group and bacterial abundance, h) Association analysis in MaAsLin2 between S1 group and bacterial abundance, i and j) MaAsLin2 generated graph of *L. reuteri*, k and l) MaAsLin2 generated graph of *Candidatus* Gallimonas intestinalis. *N* = 80 (20 in each group with 10 male and 10 female). (*P < 0.05); ***P < 0.001). Alpha diversity was analysed with 2-way ANOVA and Tukey's test, while beta diversity was assessed using a 2-sided Wilcoxon rank-sum test with Benjamini-Hochberg correction.

For taxonomic analysis, to capture the full diversity of WHS mice gut microbiome, we used the full NCBI/RefSeq prokaryote genome sequence database (NCBI RefSeq Complete V205)^46^. We identified 665 bacterial species spanning 11 phyla, 21 classes, 45 orders, 103 families, and 339 genera (Fig. 3a). Of these, 427 species were common to all 4 groups and contributed 99.8% abundance in all samples (Fig. 3b). In terms of alpha diversity, we observed similar Shannon diversity across all groups (Fig. 3c). Taxonomic beta diversity, calculated using Bray-Curtis dissimilarity, also showed no significant differences between the groups (Fig. 3d). However, when comparing the mean Bray-Curtis distance, we found a significant difference between groups C1 and S2 (*P* < 0.01) (Fig. 3e). Overall, these findings suggest that host selection pressure for tameness behaviour does not consistently affect the overall taxonomic diversity of the gut bacterial microbiota in mice.

We conducted Pearson correlation analysis on the tameness parameters scores with 40 significant taxa identified from the random forest analysis. This analysis highlighted that several *Lactobacillus* bacteria showed a significant positive correlation with both active and passive jumping behaviours, parameters related to wildness, while showing a significant negative correlation with active heading. On the other hand, *Bacteroides* sp. CBA7301, *Ligilactobacillus animalis*, and *Prevotella bivia* were significantly positively correlated with our selection pressure- active contacting (Fig. 3f).

Our metagenomic analysis identified 410 083 gene families across all samples, with 240 161 shared among all 4 groups, indicating a core set of functionalities. Each group also displayed unique gene families, reflecting the functional diversity of the gut microbiota (Fig. S3A). Chao1 richness analysis showed the C1 group had the highest functional richness and significantly more than the S1 group (*P* < 0.01) (Fig. S3B). In contrast to taxonomic diversity, Shannon diversity in the S1 group was significantly lower than in the C1 group (Fig. S3C), but beta diversity did not differ (Fig. S3D). Using the MetaCyc database, we identified 378 gut microbial pathways, with 263 common to all groups (Fig. S3E). Association analysis via MaAsLin2 revealed 88 pathways in the S2 group and 49 in the S1 group that met the *q*-value cut-off, with 35 showing consistent trends (Table S4). Pathways related to amino acid biosynthesis and energy generation were enriched in the selected groups, suggesting that selection for tameness may affect these metabolic functions and contribute to behavioural traits.

### 3.3. The selected groups exhibited higher abundance in *L. reuteri*

To identify the enrichment of bacterial species in tame mice and make the association more robust, we compared each selected group with its respective control group. For this analysis, we used MaAsLin2 to associate species-level gut microbiome composition with the WHS mouse group (Fig. 3g and h). For MaAsLin2 analysis, only species that were significantly associated in both groups were considered to differ in abundance. We found that *Limosilactobacillus reuteri* (Fig. 3i and j), previously known as *Lactobacillus reuteri*, one of the lactic acid bacteria, and *Candidatus* Gallimonas intestinalis (Fig. 3k and l) were enriched in both selected groups compared with their respective controls.

Among the 374 MAGs generated, 1 high-quality MAG from *L. reuteri* and 6 MAGs of *Candidatus* Gallimonas genus were obtained. To quantify gut bacterial abundance, we used CoverM for mapping reads to all MAGs, with mapping rates ranging from 75% to 93% (Fig. S4A). Similar to Fig. 3 (the Kraken2 results), we found that *L. reuteri* was significantly enriched in both selected groups compared with nonselected groups (S1, *P* < 0.05; S2, *P* < 0.001) (Fig. S4B and C). However, the relative abundance of the 6 *Candidatus* Gallimonas MAGs did not show a significant difference between the selected and nonselected groups (Fig. S4D–I). These results combined with those of the Pearson correlation analysis performed earlier (Fig. 3f) indicate that *L. reuteri* is the only bacterium that is consistently associated with tameness across our analyses. This association suggests that *L. reuteri* is linked with increased active tameness in the selected groups.

### 3.4. The selected groups have a higher level of plasma pyruvate

**Fig. 4.**
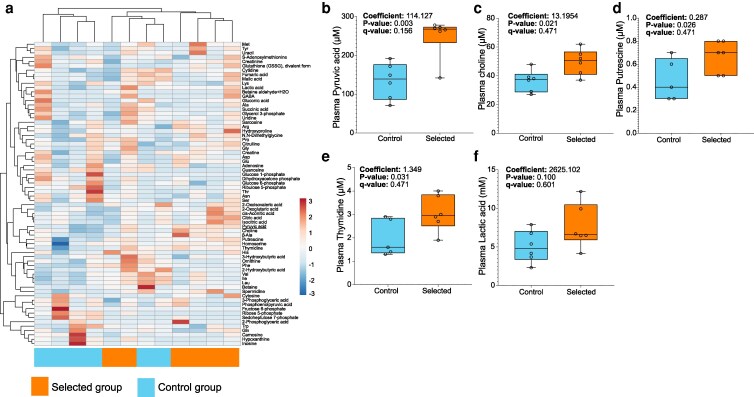
Pyruvic acid is significantly high in selected mice. a) Heatmap with hierarchical clustering using Pearson correlation distance of 70 metabolites identified, b) Plasma pyruvic acid, c–e) Significantly higher metabolites before FDR correction in MaAsLin2, c) Plasma choline, d) Plasma putrescine, e) Plasma thymidine, f) Plasma lactic acid, *N* = 12 (6 in each group).

The influence of the gut microbiota on animals occurs through the production of metabolites that are absorbed into the host and change its behaviour. To identify the metabolites that may contribute to changes in tameness, we conducted a metabolomic analysis of plasma samples obtained from WHS mice. From the capillary electrophoresis time-of-flight mass spectrometry (CE-TOFMS) measurements, 281 peaks (179 in cation mode and 102 in Anion Mode) were detected and annotated according to HMT's standard library and the known-unknown peak library. Among the target metabolites, 70 (46 in cation mode and 24 in Anion Mode) were detected and quantified (Table S5). Correlation clustering of 70 metabolites did not show any visible difference between the control and selected groups (Fig. 4a). Of these, 4 metabolites were significantly higher in selected mice (Fig. 4b–e). To strengthen our findings, we performed MaAsLin2 analysis and after FDR correction, only pyruvic acid remained significantly associated with selected mice (TableS5). The plasma lactic acid concentration, which maintains homeostasis with pyruvic acid in the body, was also observed and found to be not significantly different between the selected and control groups (Fig. 4f).

### 3.5. *L.* *reuteri* administration increases tameness behaviour in nonselected mice

**Fig. 5.**
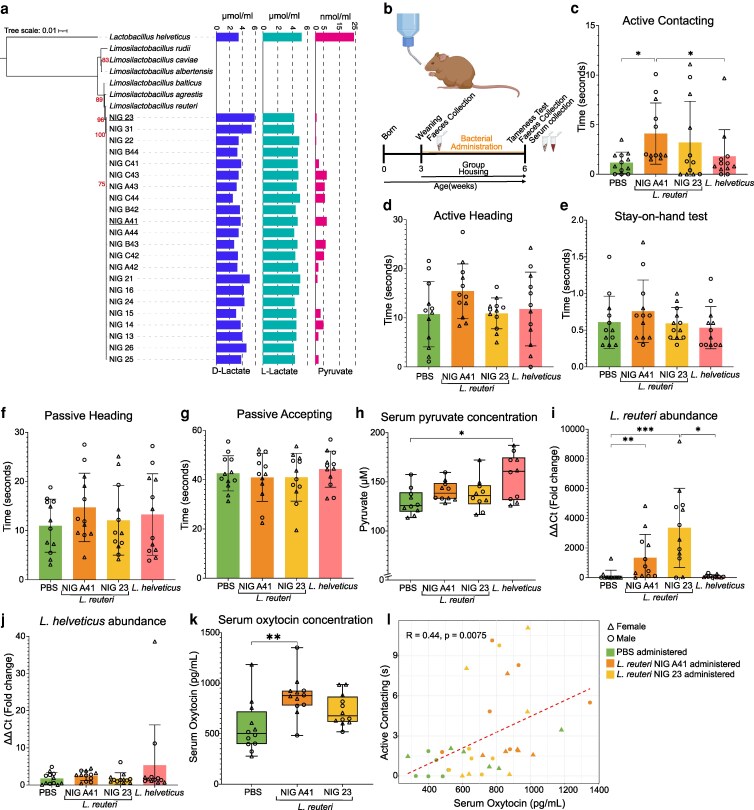
*Limosilactobacillus reuteri* administration can increase tameness behaviour in mice. a) Maximum likelihood tree of 16S rDNA region; different species of *Limosilactobacillus* genus was used for identification and *L. helveticus* was used as outgroup. 1000 bootstrap replicates were performed, and bootstrap values greater than 70% are shown next to the corresponding branches. Bar graph represent pyruvate and lactate secretion in GAM media by different colonies of *L. reuteri* isolated in current study and *L. helveticus*, b) scheme of bacteria administration to control mice through drinking water; c–g) Different tameness test parameters after bacterial administration, c) Active contacting, d) Active heading, e) Stay on hand test, f) Passive heading, g) passive accepting, h) Serum pyruvate level after bacterial administration, i) qRT-PCR quantification of *L. reuteri* present in faeces; j) qRT-PCR quantification of *L. helveticus* present in faeces; k) Serum oxytocin concentration, l) Pearson correlation between oxytocin concentration and active contacting time. *N* = 48 (12 in each group with 6 male and 6 female) in c–g, i, and j), *N* = 40 (10 in each group with 5 male and 5 female) in h), and *N* = 36 (12 in each group with 6 male and 6 female) in k and l). (*P < 0.05; **P < 0.01; ***P < 0.001). Bar graphs show means ± SD with individual data points. Normality of tameness test data was assessed with the Shapiro–Wilk test. One-way ANOVA with Tukey's test was used for normally distributed data, and the Kruskal–Wallis test with Dunn's test was used for non-normal data. Oxytocin concentration, daily water intake, and serum pyruvate were analysed using 2-way ANOVA followed by Tukey's test.

Our gut microbiome analysis revealed no significant differences in overall taxonomic or functional diversity between the selected and nonselected mouse groups, but identified a consistent enrichment of a specific bacterial species, *L. reuteri*, in the tame mice. These findings prompted us to focus on the potential contribution of this specific bacterium, rather than broader community-level effects, in subsequent functional analyses. To examine the effects of *L. reuteri* and pyruvate in mice, strains that secrete more and less pyruvate were isolated and administered through drinking water. We isolated the bacterium from caecum material and faeces of the selected group of WHS mice. We isolated 22 *L. reuteri* strains using a specialized media with raffinose and found that 16 colonies secreted pyruvate into GAM culture media via biochemical assays (Fig. 5a). *Lactobacillus helveticus* JCM1120, a pyruvate-secreting bacterial species^86^, was used as a positive control of pyruvate secreting strain. Additionally, we assessed D-lactate and L-lactate secretion due to their homeostatic relationship with pyruvate (Fig. 5a). In the experiment of bacterial strain administration to C1 group mice, we chose NIG-A41 (high-pyruvate secreting strain), NIG-23 (low-pyruvate-secreting strain), as well as the *L. helveticus* JCM1120. After administration of the cultured bacterial strains for 21 d through drinking water to nonselected C1, tameness tests were conducted (Fig. 5b). Mice treated with pyruvate secreting *L. reuteri*, NIG-A41, showed significantly increased active tameness compared with the PBS-administered group (*P* < 0.05) and *L. helveticus* administered group (*P* < 0.05) (Fig. 5c). Although not statistically significant, mice treated with less pyruvate-secreting strain, NIG-23, showed higher levels of active tameness compared with the mice treated with PBS. However, mice treated with *L. helveticus* did not show elevation in the active tameness compared with the PBS-treated mice. Active heading was also higher in NIG-A41 treated group but the difference was not significant (Fig. 5d). Other behavioural parameters, stay-on-hand test, passive heading, and passive accepting did not show prominent increase in NIG-A41 treated group (Fig. 5e–g). Serum pyruvate level was higher in *L. helveticus* treated group (*P* < 0.05) but neither NIG-A41 nor NIG-23 treated mice showed any difference in serum pyruvate levels compared with the PBS treated group (Fig. 5h). We also examined the colonization of these bacterial strains in host mice by qPCR analysis of the bacterial DNA obtained from faeces. Significantly higher levels, more than 1500 times in average, of the *L. reuteri* genomes were found in the faeces of both NIG-A41 (*P* < 0.01) and NIG-23 (*P* < 0.001) treated mice compared with the PBS-treated mice (Fig. 5i). In contrast, the mice treated with *L. helveticus* JCM1120, which was originally isolated from Emmental (Swiss) cheese^87^, did not show significant increase in the mice faeces (Fig. 5j). We also monitored body weight changes and water intake during the administration period, but no significant differences among the groups were observed (Fig. S5A–D). Because a previous study reported that the daily administration of *L. reuteri* results in increased blood oxytocin levels^88^, we examined the oxytocin levels in these mice. Serum oxytocin levels were significantly higher in mice treated with NIG-A41 (*P* < 0.01) compared with those in the PBS-treated group, but there was no significant difference in the mice treated with NIG-23 (Fig.5k). In addition, there is a significant mild Pearson correlation between serum oxytocin concentration and active contacting time (*R* = 0.44, *P* = 0.0075) (Fig. 5l). These results showed that the long-term administration of a specific strain of *L. reuteri*, NIG-A41, resulted in a higher level of active tameness with higher blood oxytocin levels, as well as higher number of faecal *L. reuteri*.

### 3.6. Plasmid-encoded gene repertoires differ markedly between the 2 *L. reuteri* strains despite near-identical chromosomal backgrounds

**Fig. 6.**
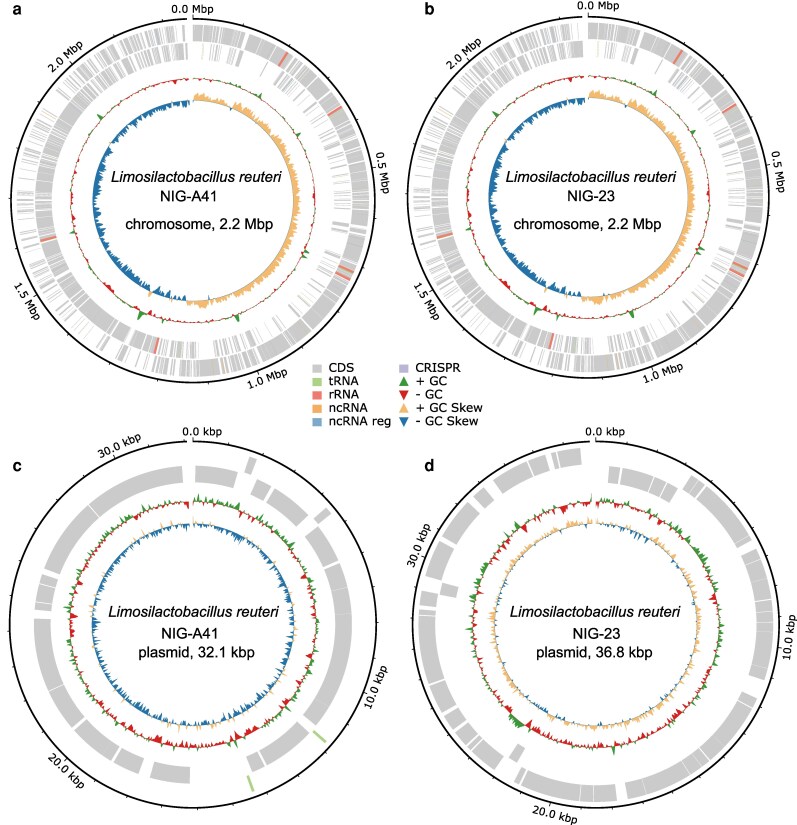
Genome architecture of *Limosilactobacillus reuteri* strains NIG-A41 and NIG-23. Circular representation of the chromosomal genomes of *L. reuteri* NIG-A41 a) and NIG-23 b). Circular representation of the plasmid genomes of NIG-A41 c) and NIG-23 d). In each panel, the innermost circle represents GC skew, indicating the imbalance between guanine (G) and cytosine (C) across the genome. Positive GC skew values are plotted outward from the baseline, whereas negative GC skew values are plotted inward. The transition points between positive and negative skew likely correspond to the replication origin and terminus. The second inner circle shows GC content. Regions with GC content higher than the genome average are plotted outward from the baseline, whereas regions below the average are plotted inward. The outermost circles display genome annotations, including coding sequences, tRNA, rRNA, ncRNA, regulatory ncRNA regions, and CRISPR elements. Features encoded on the positive strand are shown on the outer track, whereas those on the negative strand are shown on the inner track.

**Fig. 7.**
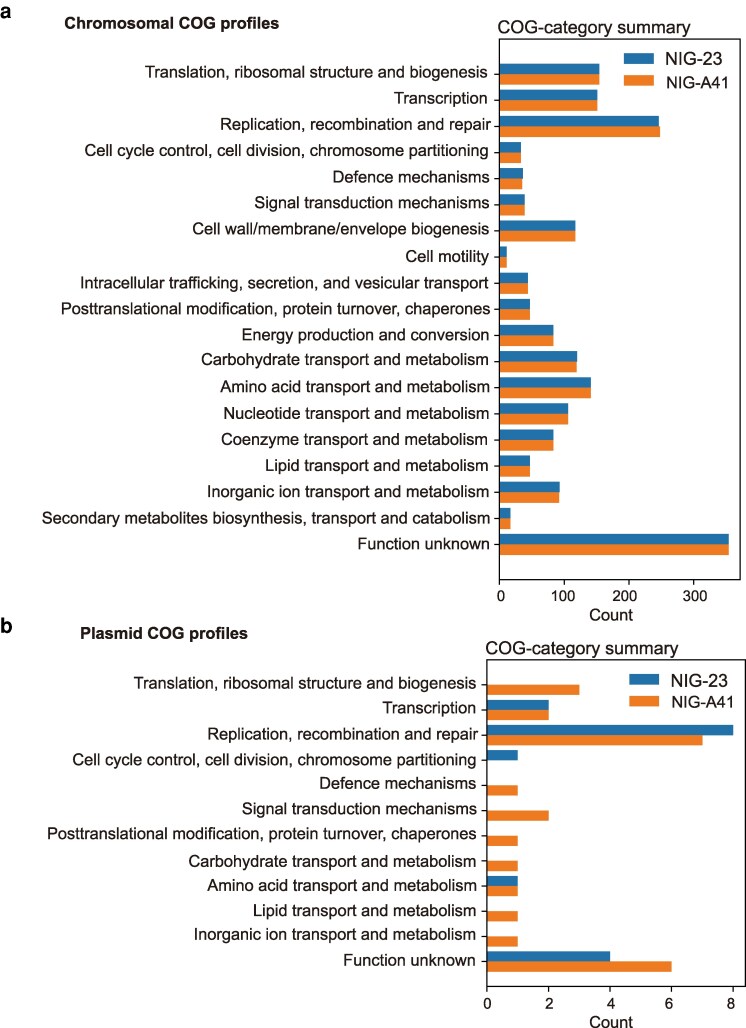
Comparison of COG functional categories between NIG-A41 and NIG-23. a) Chromosomal and b) plasmid gene counts assigned to COG functional categories in strains NIG-23 and NIG-A41. Bar plots indicate the number of genes in each COG category. Chromosomal profiles (a) show similar distributions between the 2 strains across all categories. In contrast, plasmid profiles (b) show differences in gene counts among several COG categories between the strains.

Oral administration of live *L. reuteri* strain NIG-A41 for 3 wk resulted in a statistically significant increase in plasma oxytocin levels relative to mice receiving NIG-23. In parallel, behavioural analysis indicated that mice given NIG-A41 displayed greater propensity to approach a human hand, consistent with enhanced tameness. Whole-genome sequencing revealed that the chromosomal DNA of NIG-A41 and NIG-23 shared 99.9911% nucleotide identity and harboured nearly identical chromosomal gene repertoires (Fig. 6a, b and Table S6). To further compare functional repertoires, we performed COG-based annotation of both chromosomal and plasmid-encoded genes. As expected, chromosomal COG profiles were nearly indistinguishable between NIG-A41 and NIG-23 (Fig. 7a), consistent with their highly conserved genomic backgrounds. In contrast, plasmid-encoded gene repertoires showed clear functional divergence (Fig. 7b). The plasmid of NIG-23 largely comprised a subset of the functional categories found in NIG-A41, with a modest enrichment in genes related to replication, recombination and repair, and cell cycle-associated processes. By contrast, the plasmid of NIG-A41 spanned a broader range of functional categories, including genes involved in metabolism, cellular processes and signalling, and information storage and processing. Several COG categories were uniquely detected in NIG-A41, pointing to an expansion of functional capacity at the plasmid level. In addition, NIG-A41 contained a higher proportion of poorly characterized genes, suggesting the presence of uncharacterized functional elements.

## 4. Discussion

In this study, we investigated the relationship between gut microbiota and active tameness behaviour and explored its potential underlying mechanisms. We studied the gut microbiota in selected groups of mice that exhibited high tameness and in the nonselected groups of mice that exhibited low tameness. Our findings reveal that while selection for tameness does not markedly change the taxonomic or functional diversity of the gut microbiota, but leads to the enrichment of 1 specific bacterial species, *L. reuteri*, in tame mice. Furthermore, our plasma metabolic analysis revealed a significant elevation of pyruvate levels in tame mice. The administration of pyruvate-secreting *L. reuteri* increases blood oxytocin levels and active tameness in nonselected mice.

A key strength and conceptual advance of this study lies in the use of a selectively bred wild-derived heterogeneous stock (WHS) mouse system, in which selection was applied exclusively to a single behavioural trait, active tameness, while maintaining a shared genetic background and controlled environmental conditions^20^. By comparing 2 independently selected high-tameness groups with 2 nonselected control groups derived from the same founder population, this experimental design minimizes confounding effects arising from genetic divergence, environmental variation, and differences in husbandry that commonly affect microbiome studies. This approach provides a unique and controlled framework to investigate the contribution of the gut microbiota to domestication-related behavioural traits.

We did not observe a consistent difference in gut bacteriome alpha or beta diversity between control and selected mice, suggesting that selective breeding for tameness does not influence these diversity metrics (Fig. 3). At the species level, several studies have associated bacterial genera and species with domestication traits in animals. Laboratory mice show higher levels of *Akkermansiaceae*, *Streptococcaceae*, and *Enterobacteriaceae* than wild types^11^. Domesticated horses exhibit more archaea than feral ones^13^, while domesticated buffalo have enriched Cyanobacteria and TM7 phyla^14^. In chickens, low fear selection increases *Clostridiales* and *Bacteroidales*, while high fear boosts *Lactobacillales* population^16^. These reports suggested that a factor other than host genetics, the gut microbiota, is associated with the domestication process. Although fully isolating experiments from external influences is challenging, uniform conditions for WHS mice helped mitigate this issue, revealing a link between tameness selection and *L. reuteri* abundance (Fig. 3).

The gut microbiota can alter host energy and lipid metabolism by producing energy metabolites, such as pyruvate, fumaric acid, and citric acid, and by influencing triglyceride levels in the host plasma^89^. Pyruvate originating from the gut microbiome is absorbed by the host and contributes to the priming of the immune system and protection against *Salmonella* infection. This pyruvate is secreted by *L. helveticus*^86^, a species which belongs to the same family (*Lactobacillaceae*) as *L. reuteri*. High circulating pyruvate levels in the blood and brain may also protect against Alzheimer's disease^90^ by reducing age-related cognitive decline^91^. In the present study, we found that mice with high active tameness had high plasma pyruvate levels (Fig. 4b).Twenty-two strains of *L. reuteri* were isolated by culturing colonies on selective media, sixteen of which showed pyruvate secretion into GAM medium, which is a novel finding^86^. Previous studies have linked *L. reuteri* administration to altered social behaviours in mice^92–94^. We found that bacteria isolated from the same host species exhibited more optimized colonization, without the sex-based colonization differences reported by Donovan et al.^94^. Administration of *L. reuteri* through drinking water surged the populations over 1500-fold (Fig. 5i), and this large change of *L. reuteri* levels likely contributed to the observed increase in tameness.

Our results underscore the host genetics’ pivotal role in tameness, with C1 mice showing a less pronounced tameness level after *L. reuteri* administration (Fig. 5c) compared with S1 and S2 groups (Fig. 1g). This indicates that while the gut microbiota may influence behaviour, changes in host genetic factors are a major factor for changes in tameness during the selective breeding. Despite these facts, the result that long-term administration of live *L. reuteri* via drinking water can significantly increase tameness behaviour by vastly increasing its population in the gut is noteworthy for future studies.

The role of *L. reuteri* in influencing tameness behaviour warrants further investigation to elucidate the underlying mechanisms. Previous research by Matsumoto et al.^95^ indicated that in WHS mice selected for tameness, there is higher expression of the gene for the oxytocin receptor–a receptor for the social bonding neuropeptide hormone, oxytocin, in the hippocampus. In addition, tameness selection was found to lead to increases in sociability in WHS mice^82^. Similarly, a previous study observed an increase in plasma oxytocin levels in C57BL/6 mice treated with *L. reuteri* through their drinking water^88^. The administration of *L. reuteri* was also associated with an increase in oxytocin-positive neurons in the paraventricular nucleus of the hypothalamus^92,93^. Sgritta *et al*.^96^ demonstrated that *L. reuteri* communicates with the brain, thereby affecting social behaviour via the vagus nerve. Buffington et al.^97^ identified that the gut microbiota metabolite tetrahydrobiopterin, enhanced by *L. reuteri*, can alleviate social deficits in germ-free mice, a process linked to increased oxytocin release^98^. Danhof *et al*.^99^ discovered that oxytocin is produced and secreted by intestinal epithelium enterocytes, triggered by *L. reuteri*. These collective findings pave the way for a hypothesis that oxytocin might be a central player in how *L. reuteri* influences tameness behaviour. In our study, basal oxytocin levels were higher in the selected groups compared with the nonselected groups, and *L. reuteri* administration boosted serum oxytocin in the nonselected (C1) mice (Fig. 5k). However, at present, our data do not provide direct evidence demonstrating a causal relationship between oxytocin levels and active tameness. Furthermore, we were unable to establish a direct causal link between *L. reuteri* administration and the observed increase in oxytocin levels. While these findings are consistent with a potential involvement of oxytocin in the observed behavioural changes, they should be interpreted with caution, and further studies will be required to clarify the underlying mechanisms. Importantly, it should be noted that the present study does not directly assess general social behaviour, but instead focuses on active tameness, a behavioural trait that specifically reflects the motivation to approach humans and is considered a core component of animal domestication. While previous studies have primarily examined the effects of *L. reuteri* on social interactions or social deficits, our findings extend this framework by demonstrating its association with a domestication-relevant behavioural trait under controlled selective breeding conditions. This distinction is important, as it suggests that microbiota–brain interactions may contribute not only to social behaviour per se but also to behavioural changes that are central to the domestication process.

In the present study, 2 *L. reuteri* strains with nearly identical chromosomal genomes elicited markedly different effects on host oxytocin secretion and tameness. The comparison between the 2 *L. reuteri* strains highlights a clear contrast between their highly similar chromosomal backgrounds and their divergent plasmid gene repertoires. While both strains shared nearly identical chromosomal gene content and COG profiles, their plasmids differed substantially (Fig. 7a and b). In particular, NIG-A41 carried a broader and more diverse set of genes, including those related to metabolism, cellular processes and signalling, and information storage, whereas the plasmid of NIG-23 largely represented a more limited subset of these functions, with a relative bias towards genes associated with genome maintenance and mobility. This pattern suggests that the observed phenotypic differences between the strains are more likely linked to variation in plasmid-encoded functions than to chromosomal divergence. At the same time, however, we did not detect corresponding differences in plasma pyruvate levels, despite the presence of metabolism-related genes in NIG-A41. This indicates that the relationship between plasmid gene content and host phenotype is not straightforward, and may reflect the combined effects of multiple functional elements rather than a single metabolic pathway.

Pyruvate has been proposed as a biologically active microbial metabolite capable of influencing host physiology, including neuroendocrine signalling. However, our results do not support a direct causal role of pyruvate in regulating active tameness. In particular, although elevated plasma pyruvate levels were observed in the selected mice, administration of *L. reuteri* (NIG-A41) increased tameness and oxytocin levels without increasing circulating pyruvate, whereas *L. helveticus* elevated plasma pyruvate without affecting behaviour or oxytocin levels. These findings indicate that pyruvate alone is unlikely to be sufficient to induce behavioural changes and is better interpreted as a correlated metabolic marker rather than a primary mediator.

Nevertheless, we found that the *L. reuteri* strain NIG-A41, which exhibits higher pyruvate secretion than NIG-23, produced a more pronounced increase in active tameness upon administration. This strain-dependent difference represents an important finding of the present study, suggesting that functional variation among closely related bacterial strains can differentially influence host behaviour. Although the underlying mechanisms remain to be elucidated, these results highlight the importance of strain-level properties, potentially including but not limited to metabolic outputs such as pyruvate production, in modulating domestication-related behavioural traits.

Overall, our findings contribute to the growing body of knowledge about the complex relationships between behaviour, the host genome, and the gut microbiota in the context of animal domestication. Importantly, we demonstrate that strain-level differences within *L. reuteri*, particularly in plasmid-encoded gene repertoires, are associated with distinct effects on active tameness and oxytocin levels. These results highlight the importance of considering functional variation at the strain level, rather than at the species level alone, when investigating microbiota–host interactions. Furthermore, by focusing on active tameness, a key behavioural trait underlying domestication, our study contributes to the understanding of potential associations between specific microbial features and domestication-related behavioural phenotypes.

## Supplementary Material

dsag006_Supplementary_Data

## Data Availability

All data required to evaluate the conclusions of this study are presented in the paper or Supplementary Materials. All 80 raw shotgun metagenomic sequencing datasets generated in this study are available in the NCBI under BioProject PRJDB15857 with BioSample accession numbers from SAMD00614304 to SAMD00614383 and SRA accession numbers from DRR480456 to DRR480535. All 16S rDNA sequences generated in this study can be accessed using accession IDs from LC801559 to LC801580 (Please use this link to search; https://www.ncbi.nlm.nih.gov/nuccore/). The de novo genome assemblies generated in this study have been deposited in the DDBJ/ENA/GenBank databases under accession numbers AP041035 (chromosome) and AP041036 (plasmid) for the NIG-23 strain, and AP041037 (chromosome) and AP041038 (plasmid) for the NIG-A41 strain. Nonredundant set of MAGs (374) including novel ones are available in zenodo database (https://doi.org/10.5281/zenodo.8289507). This paper does not report original code. Code used in this study are available at GitHub repository (https://github.com/bhimbbiswa/Gut-microbiota-influence-on-animal-domestication).
