# Supplementary material for: Increased abundance of Limosilactobacillus reuteri in the gut of selectively bred high-tameness mice and its association with behavioural changes
Source: DNA Res. 2026 Jun 25;33(3):dsag006. doi: 10.1093/dnares/dsag006 (PMC13296792; doi:10.1093/dnares/dsag006)
Supplement: dsag006_Supplementary_Data [file dsag006_supplementary_data.zip › Supplementary_figures.pdf]

Supplementary Figures

Increased abundance of *Limosilactobacillus reuteri* in the gut of selectively bred high-tameness mice and its association with behavioural changes

Authors: Bhim B. Biswa[1,2], Hiroshi Mori[2,3], Atsushi Toyoda[4], Kazumichi Fujiwara[1], Ken Kurokawa[2,5], Tsuyoshi Koide[1,2] *

[1]Mouse Genomics Resource Laboratory, National Institute of Genetics, Mishima, Shizuoka, Japan
[2]Graduate Institute for Advanced Studies, SOKENDAI, Mishima, Shizuoka, Japan
[3]Genome Diversity Laboratory, National Institute of Genetics, Mishima, Shizuoka, Japan
[4]Comparative Genomics Laboratory, National Institute of Genetics, Mishima, Shizuoka, Japan
[5]Genome Evolution Laboratory, National Institute of Genetics, Mishima, Shizuoka, Japan

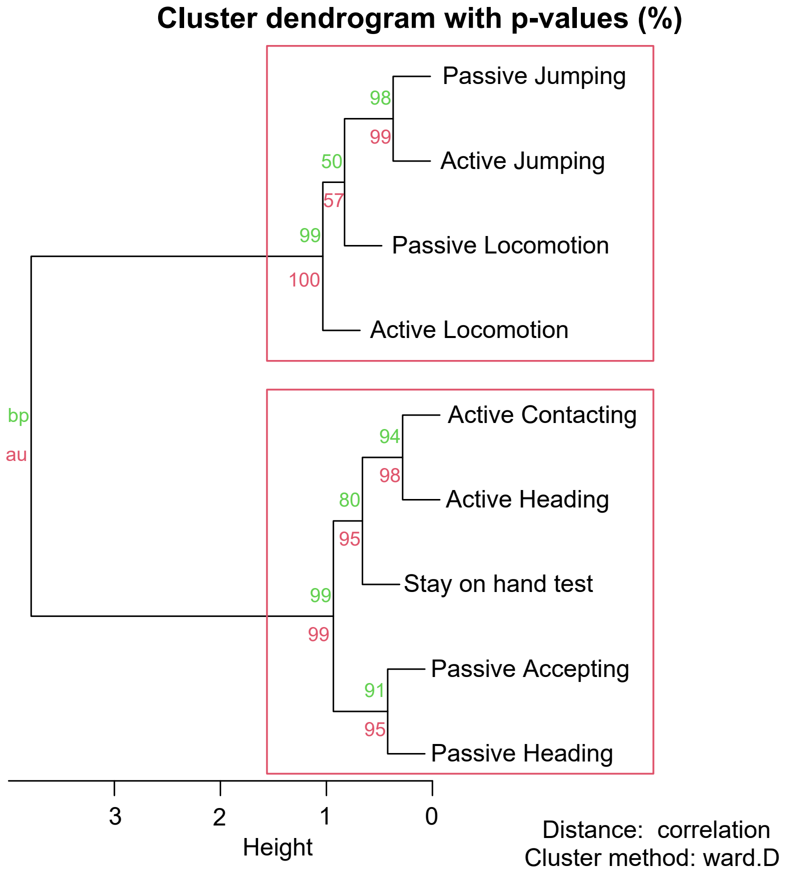

Figure S1: Correlation clustering of tameness test parameters.
All nine parameters were normalized and clustered using the correlation distance and Ward's method. The red rectangle represents significant clusters based on the p-value calculated using pvclust. 'au' stands for (Approximately Unbiased) p-value and 'BP' for Bootstrap Probability values.

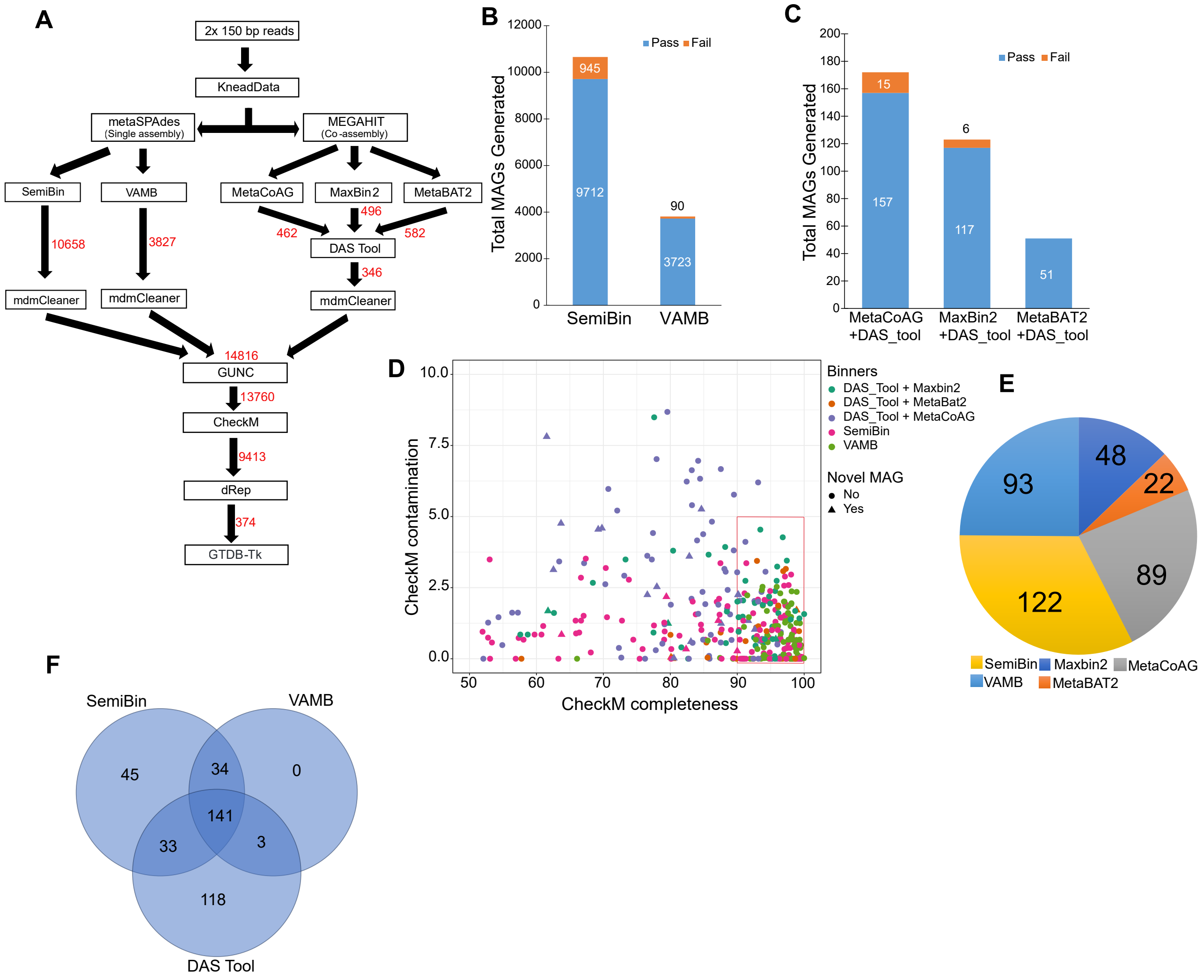

Figure S2: Details of MAGs generated.
(A) Scheme for high quality metagenome assembled genome (MAGs) generation. The number of MAGs generated at each step is shown in red. (B-C)Total MAGs generated by each binner, including the number of MAGs passing the GUNC chimerism test. (D) CheckM result of all 374 MAGs obtained, red rectangle ecompasses MAGS which have >90% completness and <5% contamination, (E) Distribution of origin of 374 MAGs generated in the current study.
(F) Venn diagram of origin of MAGs from same cluster of dRep (These data were compiled from clustering result of dRep where a MAG is considerd to be of the same species if they cluster together at 95% ANI).

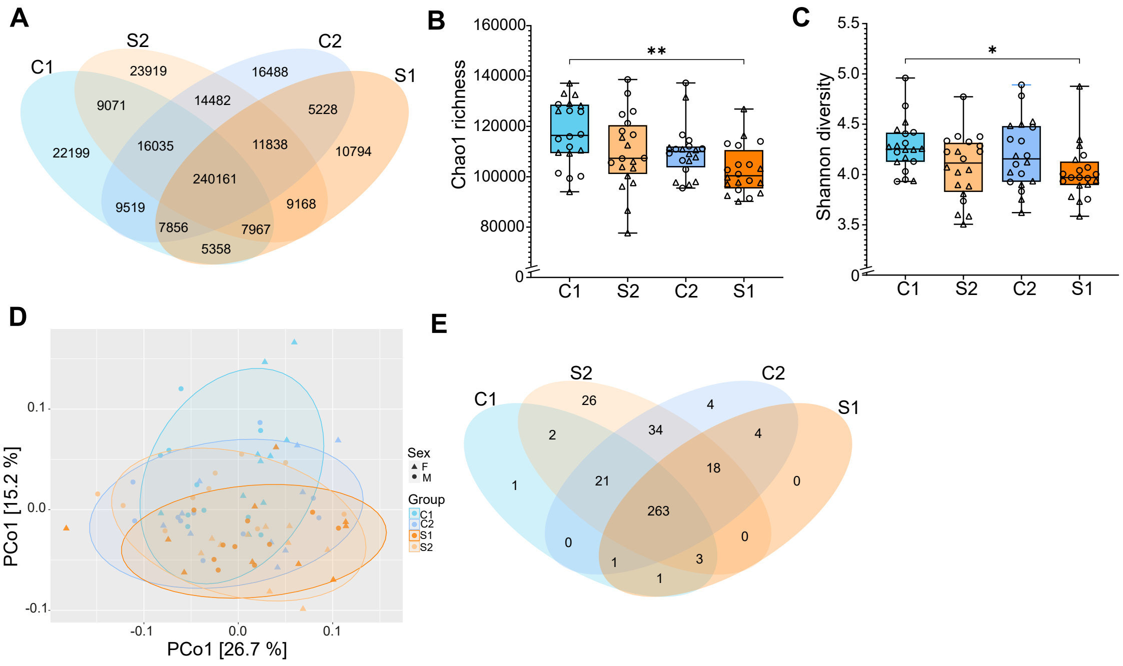

Figure S3: Host tameness selection pressure does not change gut microbiota functional diversity
(A) Venn diagram showing all identified gene family; (B) gene family Chao1 richness, (C) Gene family Shannon diversity; (D) Beta diversity based on Bray–Curtis dissimilarity. (E) Venn diagram of gut microbiota metabolic pathways identified. N= 80 (20 in each group with 10 males and 10 females). (*p<0.05); **p<0.01; ***p<0.001).

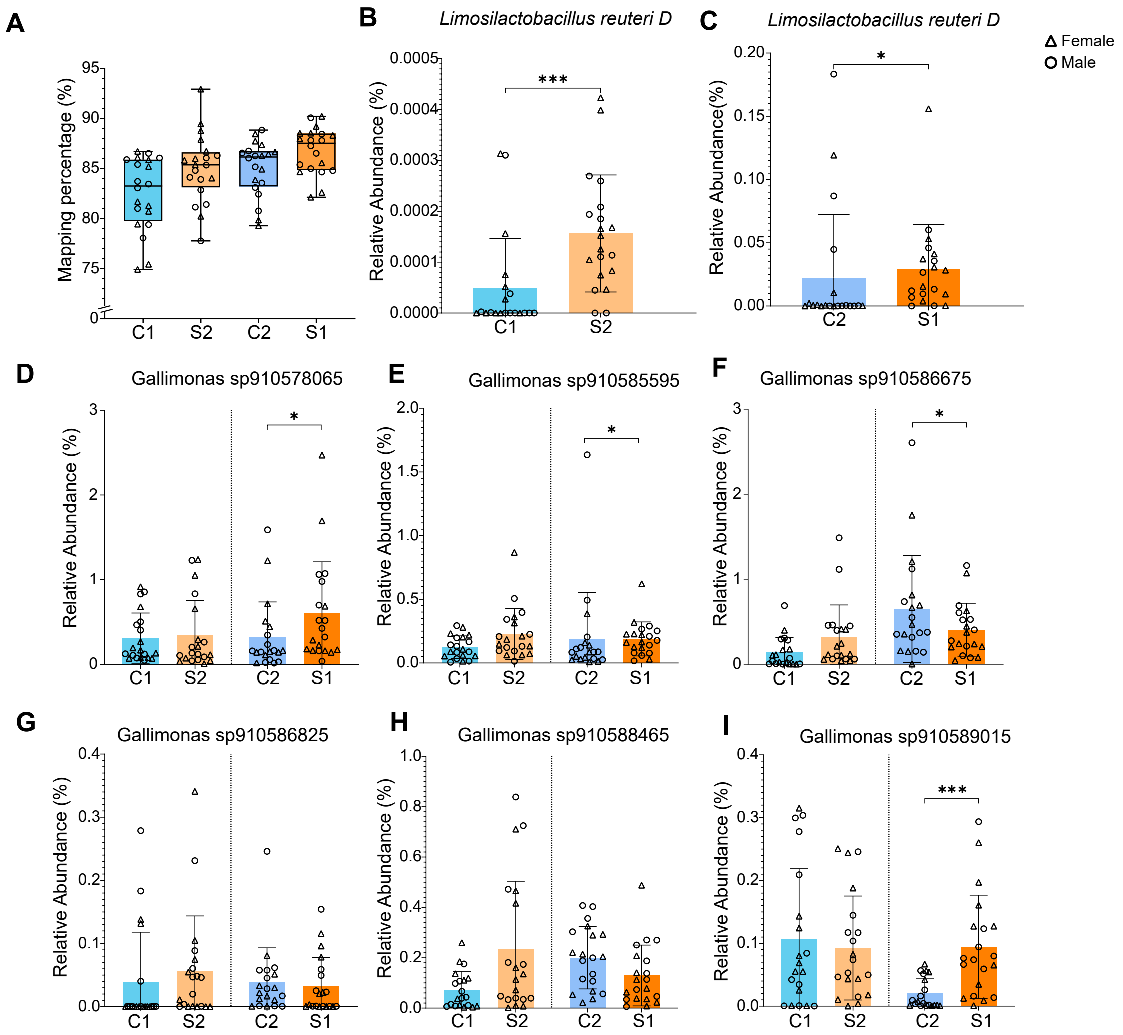

Figure S4: Relative abundance of MAGs.

(A) Mapping percentage of filtered sequences from each sample against MAGs generated in this study; (B) relative abundance of *Limosilactobacillus reuteri* MAGs in C1 & S2 (One sample was not shown as it exceeds y-axis, but utilized for statistical calculation); (C) relative abundance of *Limosilactobacillus reuteri* MAGs in C2 & S1; (D-I) relative abundance of six *Candidatus* Gallimonas MAGs; N= 80 (20 in each group with 10 male and 10 female). (*p<0.05); **p<0.01; ***p<0.001). Bar graphs show means ± the SD with ndividual data points.

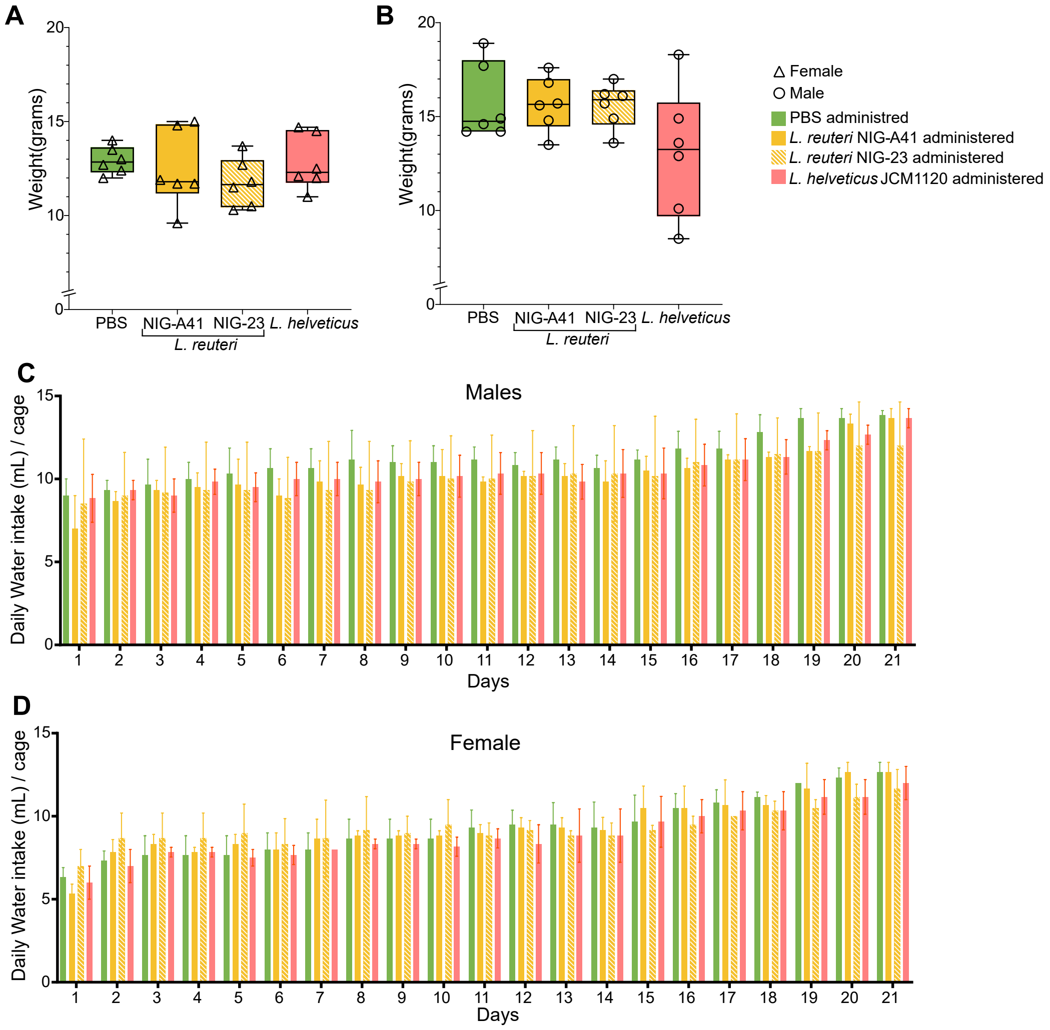

Figure S5: No change in body weight and water intake during bacterial administration.
(A) Body weight of females at 6 week of age after bacterial administration (B) Body weight of males at 6 week of age after bacterial administration; (C) Daily water intake in males for 21 days; (D) Daily water intake in female for 21 days. N=48 (12 per group). As mice were kept as same sex pairs during this experiment, water intake data were obtained from six cages per group (3 male and 3 female cages). Bar graphs show means ± SD with individual data points.
