# Supplementary material for: Increased abundance of Limosilactobacillus reuteri in the gut of selectively bred high-tameness mice and its association with behavioural changes
Source: DNA Res. 2026 Jun 25;33(3):dsag006. doi: 10.1093/dnares/dsag006 (PMC13296792; doi:10.1093/dnares/dsag006)
Supplement: dsag006_Supplementary_Data [file dsag006_supplementary_data.zip › Supplementary_methods.pdf]

Supplementary Methods

Increased abundance of *Limosilactobacillus reuteri* in the gut of selectively bred high-tameness mice and its association with behavioural changes

Authors: Bhim B. Biswa[1,2], Hiroshi Mori[2,3], Atsushi Toyoda[4], Kazumichi Fujiwara[1],

Ken Kurokawa[2,5], Tsuyoshi Koide[1,2] *

[1]Mouse Genomics Resource Laboratory, National Institute of Genetics, Mishima, Shizuoka, Japan

[2]Graduate Institute for Advanced Studies, SOKENDAI, Mishima, Shizuoka, Japan

[3]Genome Diversity Laboratory, National Institute of Genetics, Mishima, Shizuoka, Japan

[4]Comparative Genomics Laboratory, National Institute of Genetics, Mishima, Shizuoka, Japan

[5]Genome Evolution Laboratory, National Institute of Genetics, Mishima, Shizuoka, Japan

Animal housing conditions and mouse maintenance.
The mice used in our experiments were bred and maintained in SPF conditions within the animal facility at NIG. All equipment used for animal maintenance, including standard-sized transparent polycarbonate cages, cage tops, paper bedding materials, and other essential supplies, underwent sterilization through autoclaving. Mice were weaned at approximately 3–4 weeks of age and were co-housed with their same-sex littermates until the tameness test was conducted. During cage changes and behavioural tests, each mouse was gently handled by its tail using tweezers covered with a silicone tube to minimize stress. All personnel involved in the experiments wore polyester suits, non-woven fabric masks, and double gloves (cloth gloves under latex gloves) to maintain a sterile environment. In all experiments, we took careful measures to prevent microbiome cross-contamination. Cages housing mice from different groups were not placed in close proximity to each other. This precaution ensured that the microbial environments of each group remained distinct and uncontaminated by other groups.

Selective breeding of WHS
WHS mice were generated by crossing eight wild strains, BFM/2Ms, PGN2/Ms, HMI/Ms, NJL/Ms, BLG2/Ms, KJR/Ms, CHD/Ms, and MSM/Ms, derived from different countries to expand the genetic heterogeneity[1,2]. The founder stock, maintained with 16 breeding pairs, was split into two groups in the third generation to create S1 and C1, and, henceforth, S1 was selectively bred for higher active tameness. In the fifth generation, another selected group, S2, was split from C1, and another non-selected group, C2, was split from S1. Thereafter, S2 was selectively bred for a higher level of active tameness and C2 was maintained as the non-selected group. Mice used for breeding were selected from the five female and five male offspring of each pair based on the highest contacting score in the active tameness test, and if two or more animals showed the same highest contacting score, the individual with the higher heading score in the same test was selected.

Mice faeces collection
In all experiments, mouse faecal samples were collected following the tameness test, ensuring consistency in timing between 17:00 and 18:00 on the same day. To maintain sterility and minimize environmental variables, the test mice were housed in autoclaved, sterile cages devoid of any bedding material. This setup allowed for natural defecation without external prompting. The faeces were carefully collected in an Eppendorf tube using sterile aluminium foil and immediately stored at -80˚C.

Blood serum and plasma collection
Blood plasma was obtained when mice reached 11 weeks of age. For the collection procedure, the mice were first anaesthetized using an intraperitoneal (IP) injection of pentobarbital at a dosage of 50mg/kg body weight (Tokyo Chemical Industry Co. Ltd., Japan). Following anaesthesia, blood samples were drawn via cardiac puncture using a syringe pre-treated with heparin to prevent coagulation. The collected blood was then transferred into heparinized Eppendorf tubes. These tubes were allowed to stand undisturbed at room temperature for 30min to enable the separation of blood components. Subsequently, the tubes were centrifuged at 10,000G for 10min at a temperature of 4°C. For serum isolation, the same protocol was used, excluding heparin treatment.

Metagenomic DNA isolation from mice faeces
In our modified approach to metagenomic DNA isolation, we utilized protocol #6 from Costea et al.[3], incorporating the QIAamp Fast DNA Stool Mini Kit (QIAGEN GmbH, Hilden, Germany) for DNA extraction. The process began with treating 180–220mg of faecal samples

with 1ml of lysis buffer (500mM NaCl, 50mM Tris-HCl at pH 8.0, 50mM EDTA, and 4% sodium dodecyl sulfate). This mixture was homogenized for 5min using a handheld homogenizer (Leda Trading Corp.), followed by the addition of 10µl proteinase K and sterile zirconia beads (1.0mm, 20-30; BioSpec, Inc., USA). For thorough mixing, the sample was vortexed at maximum speed for 10 min using a Vortex-Genie 2 mixer (MO BIO Laboratories, Inc., USA) and then incubated at 95°C for 15min. Post-incubation, the sample was centrifuged at 16,000xg for 5min at 4°C, and the supernatant was transferred to a new 2mL tube. The remaining pellet was subjected to a second round of lysis, resuspended in 300µL of the lysis buffer, and processed as previously described. The combined supernatants from both lysis steps were then treated with 260µL of 10M ammonium acetate, followed by vortexing and incubation on ice for 5min. The solution was centrifuged at 16,000xg for 10min at 4°C, and the resulting supernatant was equally divided into two 1.5 mL tubes, each containing 750 µL isopropanol. These tubes were then incubated on ice for 30 min and centrifuged at 16,000 x g for 15 min. The supernatant was discarded, and the pellet was washed with 0.5 mL of 70% ethanol and left to air-dry. Finally, the dried pellets were reconstituted in 100 µL of TE (Tris-EDTA) buffer, and the aliquots were pooled. We added 2 µL DNase-free RNase (10 mg/mL) to the pooled DNA solution and incubated it at 37°C for 15 min. Then, 10 µL proteinase K and 200 µL buffer AL were added, vortexed for 15 s, and incubated at 70°C for 10 min. Subsequently, 200 µL of 96–100% ethanol was added to the lysate, vortexed, and passed through a QIAamp spin column, and centrifuged for 1 min. The filtrate was discarded and the column was sequentially washed with 500 µL each of Buffer AW1 and AW2. Following a final centrifugation, 100 µL AE Buffer was used to elute DNA into a new 1.5 mL tube. A spectrophotometer (NanoVue Plus) was used for the quality check. A Qubit 2.0 Fluorometer was used for precise quantification following the manufacturer's instructions. DNA integrity was checked using 0.8% agarose gel electrophoresis. The DNA was then stored at -20°C for future use.

PCR amplification of 16S rDNA

To amplify the full 1.6 kbp region of the 16S rDNA, polymerase chain reaction (PCR) was performed using the universal primers bak4 (5'-AGGAGGTGATCCARCCGCA-3') and bak11w (5'-AGTTTGATCMTGGCTCAG-3')[4,5]. The PCR cycling conditions included an initial denaturation at 95°C for 5 min, followed by 35 cycles of 95°C for 15s, 60°C for 30s, and 72°C for 2min, with a final extension at 72°C for 7min. The PCR products were then purified and subjected to Sanger sequencing.

Pyruvate, L-Lactate, and D-Lactate secretion assay

In this experiment, *L. helveticus* (JCM1120), sourced from the Japan Collection of Microorganisms (JCM), served as the positive control for pyruvate production. All bacterial strains, including the control, were cultured in Gifu Anaerobic Broth (GAM) medium (Nissui Pharmaceutical, Japan), which was supplemented with 1% glucose. The incubation was conducted for 24 h at 37°C under anaerobic conditions. To measure the concentrations of pyruvate, L-Lactate, and D-Lactate in the media, we utilized biochemical assay kits from Cayman Chemicals (Michigan, USA), strictly adhering to the manufacturer's protocol. All three assays were conducted in triplicates (technical replication).

qPCR quantification of *Limosilactobacillus reuteri* and *Lactobacillus helveticus*

Quantitative PCR (qPCR) was conducted using a Thermal Cycler Dice Real Time System III (Takara Bio Inc., Shiga, Japan). For each 25µL reaction, TB Green Premix Ex Taq II (Tli RNaseH Plus) (Takara Bio Inc., Shiga, Japan) and gene-specific primers at a concentration of 1 µM were used. The reaction mixtures included 5ng of metagenomic DNA, the quantity of

which was determined using Qubit. The cycling conditions were set at 95°C for 5min, followed by 40 cycles of denaturation at 95°C for 20s, annealing at 62°C for 20s, and extension at 72°C for 30s. After the PCR cycles, a melt curve analysis was performed in the range of 60–95°C using the default settings of the system. The primers used in the qPCR were as follows: Universal bacterial primers EUB338 (5'-ACTCCTACGGGAGGCAGCAG-3')[6], and EUB518 (5'-ATTACCGCGGCTGCTGG-3')[7]; *L. reuteri* -specific 16S-23S rRNA gene spacer primers, sg-Lreu-F (5'-GAAGATCAGTCGCAYTGGCCCAA-3'), and sg-Lreu-R (5'-TCCATTGTGGCCGATCAG-3')[8]; *L. helveticus* hsp60 specific primers, F1LHelHsp (5'-CTTTGATCGCTGATGCTATGGAAAAGGTTGGTC-3'), and R1LHelHsp (5'-GATCAACAATGACTTGCCTTGTTGAACAATTTC-3')[9]. The qPCR analysis was based on the ΔCt of the specific primers normalized to the ΔCt of the universal primers (ΔΔCt method).

Bacterial culture

All bacterial strains were cultured in MRS media for 24 h. Following this cultivation, the optical density (OD) at 590nm was measured to estimate the number of bacteria in the solution, using a standard curve. The bacteria were then washed with PBS, resuspended in PBS, and stored at -80˚C until use. We cultured a fresh batch of bacteria every week to maintain viability. For the drinking water administration and measurements, 15-mL glass test tubes stoppered with silicone caps with double ball-bearing nozzles, Drinko-measurer DM-G1 (O'hara & Co. Ltd., Tokyo, Japan), were used daily.

Mice serum pyruvate and oxytocin concentration

To measure the concentrations of pyruvate in mice serum, we utilized biochemical assay kits from Cayman Chemicals (Michigan, USA), strictly adhering to the manufacturer's protocol. Due to the limited number of sample wells, 10 randomly selected samples (5 males and 5 females) were analysed. To measure oxytocin concentration, we used Oxytocin ELISA kit (Enzo Life Sciences, USA), following manufacturer's instructions. For baseline oxytocin in WHS mice, the ELISA plate's well constraints meant we analysed 18 plasma samples (9 male and 9 female) from each group, totalling 72 samples. To evaluate the effect of *L. reuteri* treatment on blood oxytocin levels, *L. helveticus*-treated samples were excluded. Both assays were performed in duplicates (technical replication).

1. Matsumoto, Y., Goto, T., Nishino, J., et al. 2017, Selective breeding and selection mapping using a novel wild-derived heterogeneous stock of mice revealed two closely-linked loci for tameness. *Sci Rep*, **7**, 4607.
2. Matsumoto, Y., Nagayama, H., Nakaoka, H., Toyoda, A., Goto, T. and Koide, T. 2021, Combined change of behavioral traits for domestication and gene-networks in mice selectively bred for active tameness. *Genes Brain Behav*, **20**, e12721.
3. Costea, P. I., Zeller, G., Sunagawa, S., et al. 2017, Towards standards for human fecal sample processing in metagenomic studies. *Nat Biotechnol*, **35**, 1069-1076.
4. Greisen, K., Loeffelholz, M., Purohit, A. and Leong, D. 1994, PCR primers and probes for the 16S rRNA gene of most species of pathogenic bacteria, including bacteria found in cerebrospinal fluid. *J Clin Microbiol*, **32**, 335-351.
5. Goldenberger, D., Perschil, I., Ritzler, M. and Altwegg, M. 1995, A simple "universal" DNA extraction procedure using SDS and proteinase K is compatible with direct PCR amplification. *PCR Methods Appl*, **4**, 368-370.
6. Lane, D. 1991, 16s/23s rRNA sequencing. In: Erko Stackebrandt, M. G. (ed), *Nucleic Acid Techniques in Bacterial Systematics*, John Wiley & Sons, West Sussex, United Kingdom, pp. 115.
7. Muyzer, G., de Waal, E. C. and Uitterlinden, A. G. 1993, Profiling of complex microbial populations by denaturing gradient gel electrophoresis analysis of polymerase chain reaction-amplified genes coding for 16S rRNA. *Appl Environ Microbiol*, **59**, 695-700.

8. Matsuda, K., Tsuji, H., Asahara, T., Matsumoto, K., Takada, T. and Nomoto, K. 2009, Establishment of an analytical system for the human fecal microbiota, based on reverse transcription-quantitative PCR targeting of multicopy rRNA molecules. *Appl Environ Microbiol*, **75**, 1961-1969.
9. Herbel, S. R., Lauzat, B., von Nickisch-Rosenegk, M., et al. 2013, Species-specific quantification of probiotic lactobacilli in yoghurt by quantitative real-time PCR. *J Appl Microbiol*, **115**, 1402-1410.
